# Osteonevus of Nanta: A Histopathological and Morphometric Case Report of a Rare, but Otherwise Benign Lesion

**DOI:** 10.3390/reports9030198

**Published:** 2026-06-23

**Authors:** Zlatko Zlatev, Tanya Peshleevska-Vicheva, Angel Angelov, George Stoyanov, Hristo Popov

**Affiliations:** 1 Department of Pathology, Multiprofile Hospital for Active Treatment, 9700 Shumen, Bulgaria; 2 Department of Surgery, Multiprofile Hospital for Active Treatment, 9700 Shumen, Bulgaria; 3 Department of Dermatology, Multiprofile Hospital for Active Treatment, 9700 Shumen, Bulgaria; 4 Department of General and Clinical Pathology, Forensic Medicine and Deontology, Medical University—Varna, 9000 Varna, Bulgaria

**Keywords:** osteonevus, osteonevus of Nanta, osteoid metaplasia, dermal nevus, hamartoma

## Abstract

**Background and Clinical Significance**: Osteonevi, originally described by Heidingsfeld in 1908 and later by Nanta in 1911, because of whom it is known as osteonevus of Nanta, is a rare condition with not yet fully established etiopathogenesis; **Case Presentation**: Herein, we report a case of a 33-year-old female patient who presented to our institution with a papilliform pigmented lesion located on the projection of the left mandibular angle, measuring 2 × 1.5 cm. The lesion had been present since childhood; however, it had increased in size by approximately 5 mm over the previous month and had become painful. Surgical excision was performed, which went uncomplicated. Histology of the resected specimen showed a dermally based, symmetrical melanocytic proliferation, without signs of dysplasia, and an underlying keratocyst with rupture, accompanied by a surrounding foreign-body-type granulomatous reaction around inert keratin flakes. A third component of the lesion was also noted, represented by foci of osteoid and myeloid metaplasia underneath the melanocytic proliferation, without direct relation to the ruptured keratocyst. Based on the morphological findings, the diagnosis of osteonevus of Nanta was established; **Conclusions**: Oseonevus of Nanta is an extremely rare, benign morphological finding. The etiopathogenesis of these rare lesions is not yet fully established, despite several proposed mechanisms. The differential diagnosis, while typically straightforward, is broad.

## 1. Introduction and Clinical Significance

First depicted by Heidingsfeld in 1908 and later in more detail by Nanta in 1911, ossification within dermal nevi is a rare phenomenon [1,2]. The condition, trivially named osteonevus of Nanta, due to Nanta’s immense contributions to its description, is associated predominantly with conventional, non-dysplastic dermal nevi, rarely compound nevi and other non-conventional forms of intradermal nevi such as Spitz nevus, Becker nevus and dendritic forms of blue nevi (blue osteonevus) [1,3,4].

Nanta osteonevi develop predominantly in females with a predilection for facial skin, with a varied age distribution, but predominantly in younger individuals, with cases being described in the pediatric population as well [1,3,5,6]. Nanta osteonevi represent a rare group of conditions, with larger analyses of melanocytic nevi revealing that they account for around 1.5% of excised nevi, with significant predominance in the head and neck area [6]. Despite being a phenomenon of benign ossification within predominantly conventional dermal nevi, when considered in the context of cutaneous ossification, they represent nearly 20% of all cases of cutaneous ossification, independent of the nosological unit [7,8]. To date, only a single case of osteonevus of Nanta has been reported in association with malignant melanoma transformation of the lesion’s melanocytic component [9].

## 2. Case Presentation

A 33-year-old female patient presented to our institution with a papilliform pigmented lesion located on the projection of the left mandibular angle, measuring 2 × 1.5 cm. The lesion had been present since childhood; however, it had increased in size by approximately 5 mm over the previous month and had become painful. Due to the lesion’s location, size, and recent change in characteristics, surgical excision was suggested. The patient agreed and opted for further resection of a 7 × 8 mm pigmented lesion with similar characteristics, located on the left cheek, for cosmetic reasons.

Previous medical history included one pregnancy with maternal hypertension and pulmonary thromboembolism post-cesarean section at term. At presentation, the physical examination, blood work, and urinalysis were within normal reference ranges, except for a slightly elevated blood glucose concentration of 6.7 mmol/L (reference range: 3–6.2 mmol/L) and elevated neutrophils of 11.75 × 10^9^/L (reference range: 3.5–10.5 × 10^9^/L).

Excision was performed with local, infiltrative anesthesia, and the post-intervention period was uneventful.

The excised specimen sent for histology included two leaf-like skin resections, the first measuring 15 × 12 mm, with a symmetrical, exophytic, and homogeneously pigmented lesion, measuring 7 mm post-fixation. The second specimen, again a leaf-like skin excision measuring 2.8 × 2 cm, had an exophytic, homogeneously pigmented lesion measuring 1.8 × 1.2 cm post-fixation, which was symmetrical but firm on cross-section.

Histopathology of the first lesion showed a dermal melanocytic nevus, without any signs of dysplasia.

**Figure 1:**
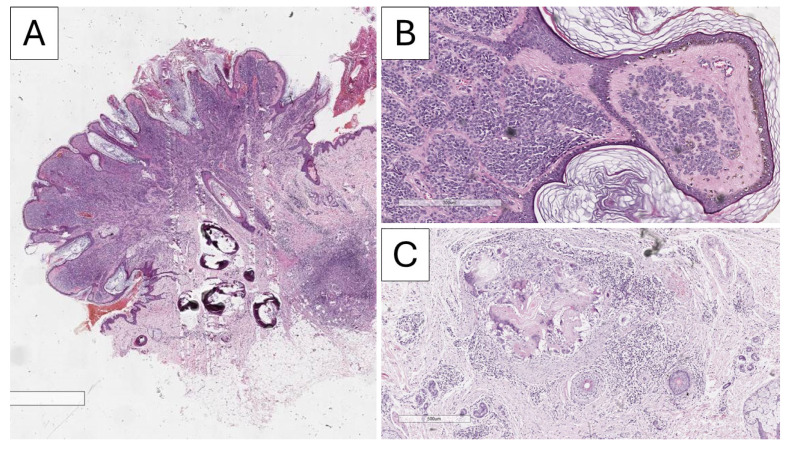
Histopathology of the lesion. (**A**): Macro slide view showing the melanocytic nevus and underlining granuloma and ossicles, H&E stain, scale bar section 2.5 mm; (**B**): melanocytic nevus without dysplastic features, H&E stain, original magnification 10×, scale bar 300 μm; (**C**): deeply located foreign-body-type granuloma surrounding keratin flakes, H&E stain, original magnification 40×, scale bar 500 μm.

**Figure 2:**
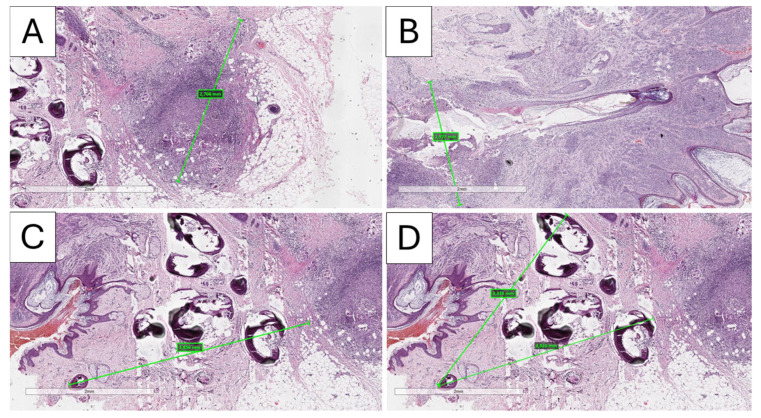
Morphometry of the components. (**A**): Morphometry of the granuloma at its greatest size, measuring 2.706 mm (green measuring line), H&E stain, original magnification 20×, scale bar 2 mm; (**B**): keratocyst with rupture and surrounding granulomatous inflammation, measuring 1.977 mm at its largest (green measuring line), H&E stain, original magnification 20×, scale bar 2 mm; (**C**): largest distance from the granuloma to the furthers away located ossicle, measuring 3.856 mm (green measuring line), H&E stain, original magnification 20×, scale bar 2 mm; (**D**): largest size of the conglomeration of ossicles, measuring 3.335 (upper) and 3.510 mm (lower) (green measuring lines), H&E stain, original magnification 20×, scale bar 2 mm.

The second lesion showed a dermally based, symmetrical melanocytic proliferation, again without signs of dysplasia, and an underlying keratocyst with rupture, accompanied by a surrounding foreign-body-type granulomatous reaction around inert keratin flakes (Figure 1). A third component of the lesion was also noted, represented by foci of osteoid and myeloid metaplasia underneath the melanocytic proliferation, without direct relation to the ruptured keratocyst (Figure 1). Spatial analysis and morphometry of the component revealed that the granuloma surrounding the ruptured cyst measured 2.7 mm at its largest, and the ruptured cyst measured 1.9 mm at its largest (Figure 2). The total size of the osteoid conglomerates measured 3.5 mm with the farthest ossicle measuring nearly 3.9 mm away from the granuloma (Figure 2). The morphometric features and specific distribution of the components suggested coexistence of the ossicles and the keratocyst rather than causation.

Based on the complex morphological findings, the diagnosis of osteonevus of Nanta with a ruptured and inflated underlying keratocyst was established.

Prior to discharge, the patient reported no pain in the area of excision, and on follow-up, inflammatory markers were within the normal range, indicating that the initial elevation of neutrophils of 11.75 × 10^9^/L (reference range: 3.5–10.5 × 10^9^/L) was likely due to the inflammatory reaction surrounding the ruptured keratocyst.

## 3. Discussion

There are three main proposed mechanisms for the etiopathogenesis of Nanta osteonevi. The first focuses on the nature and development of melanocytic nevi. By definition, non-dysplastic nevi should not be considered tumors (neoplastic) in nature but rather hamartomatous formations caused by improper migration of progenitor cells during embryogenesis and fetal development [10]. This is why several non-connected and rare conditions also bear the name of nevus, while not comprising a melanocytic proliferation, such as sebaceous nevus (Jadassohn nevus), apocrine and eccrine nevi, connective tissue (colagenoma and elastoma), as well as vascular nevi (nevus flammeus nuchae) [11]. As such, osteonevus of the Nanta may represent a rare phenomenon of hamartomatous overgrowth of two different tissue types (true hamartoma) [1,3,6].

The second proposed mechanism is that of secondary ossification in a melanocytic nevus. This mechanism focuses on two possible interactions. The first is that of chronic irritation, which would explain the premonitory nature of Nanta osteonevi of the head and neck region, leading to recurrent or chronic inflammation, which is a known causative agent for both calcification and ossification of fibrous tissue by means of connective tissue metaplastic change [1,3,6]. The second aspect of this mechanism can be explained by humoral stimulation of dermal connective tissue by factors released from the melanocytes of the nevus, predominantly transforming growth factor beta and the connective tissue growth factor [12]. This mechanism can also be combined with the previously mentioned metaplastic one, as inflammatory cells are also prominent producers of these factors [6].

The third proposed mechanism for the development of Nanta osteonevi is that of estrogen overstimulation, which would explain the more common occurrence among women [1,6]. As estrogen levels in women are higher and hyperestrogenemia is typically associated with the development of complications in the age groups in which Nanta osteonevi develop, as well as the presence of estrogen receptors on osteoblasts, overstimulation may lead to osteoid overgrowth in reactive connective tissue and hence the formation of mature bone [3,6]. This mechanism, however, seems highly unlikely due to the lack of reported aspects of hyperestrogenemia in the published cases in females, such as ovarian, endometrial and mammary malignancies. Furthermore, the presence of synchronous osteonevi is extremely rare [6].

When analyzed separately, all the proposed mechanisms appear highly unlikely on their own, except for the inflammatory one. However, inflammation has been reported as present only in a minority of Nanta osteonevi cases [6,7]. Therefore, based on the established theories so far, ossification in benign intradermal nevi is either a rare, independent phenomenon or a combination of all the proposed mechanisms.

While initially straightforward, based on its rarity and the variety of calcifying and ossifying lesions of the skin, the differential diagnosis of osteonevus of Nanta is broad. First among these is the presence of chronic irritation and inflammation, especially in cases similar to ours, where the nevus is adjacent to a ruptured keratocyst [13]. This, however, is not the case in the currently reported case, as the total area of ossification is significantly greater than the area of the keratocyst itself, as well as the significant distance from the cyst to the most distant area of calcification. Morphometric analysis, as the one performed in our case, which topically depicts the special arrangement and distance between the two components, is helpful for establishing the etiopathogenesis. While chronic inflammation is a known causative agent for dystrophic calcification, calcium deposits need to be in an intimal relationship with the foci of inflammation, which is predominantly granulomatous in such instances [14]. In our case, the farthest distance from the calcification to the keratocyst is 3.856 mm, while the largest size of the keratocyst and inflammation is 1.977 mm. The sheer defense is size, in favor of the calcifications, which makes the inflammatory mechanism for transformation unlikely. Furthermore, osteoid and myeloid metaplasia are time-dependent processes and the short timeframe (one month) of clinical evolution from symptom onset to histopathological diagnosis is relatively short, further making this mechanism unlikely. Furthermore, in most of the reported cases of Nanta osteonevi, there is no evidence of inflammation within the lesion or keratocysts present to support this mechanism as universal in this nosological unit [6].

Similarly, other keratocyst lesions and epithelial tumors may also present with calcification and osteoid metaplasia, especially in the head and neck region, such as the calcifying pilomatricoma (Malherbe tumor). The differential diagnosis for a melanocytic nevus, however, is typically straightforward [15].

Another differential is that of osteoma cutis, which can develop in the context of multiple conditions [16]. Some authors have even proposed a further, more complex mechanism for osteoma cutis as an atavistic process, especially in cases of multiple lesions [17].

Lastly, calcinosis cutis, as a broad group of conditions, can also lead to similar findings, both in its idiopathic form and in calcifilaxis forms [18]. A melanocytic component is, however, absent in these.

As already mentioned, Nanta osteonevi are both rare and have a predilection for younger individuals with a female predominance [1,5]. Occurrence has been reported in the pediatric population as well, with reports being published for children as young as two and three years old diagnosed with this rare entity [5,19,20].

While the histological spectrum of changes has been well established and most reports of Nanta osteonevi focus on the striking morphological findings, few studies have focused on its dermatoscopic characteristics. On dermatoscopy, these lesions seem to have a darker center and lighter periphery, nevus-associated broken hairs and can have a more prominent vasculature, with no specific signs pointing towards the presence of calcifications within the lesions themselves [21,22]. As such, more detailed dermatoscopic correlation needs to be studied in the future, and if possible, detailed dermatoscopic characteristics of these rare lesions should be established.

As seen in the literature, a malignant transformation of Nantas osteonevi into malignant melanoma ex osteonevus of Nanta is an exceedingly rare occurrence [9]. This, however, also warrants future research, as the osteoid component may be misinterpreted in the context of melanoma or be considered a secondary reactive finding. On the other hand, the factors that play a role in the genesis of ossicles within Nanta osteonevi may act as inhibitors of the melanoma transformation of nevocytes.

From the available literature, to the best of our knowledge, myeloid metaplasia is an exceedingly rare phenomenon, making this rare subset of Nanta osteonevi, nevi with true bone formation, myelosteonevus [5,23].

While other conditions, such as osteoma cutis, present with somewhat specific dermatoscopic features of milky white streaks, corresponding to the formation of mature bone in the cutis and subcutis, these features may be obscured in Nanta osteonevi, even in cases of significant ossification, due to the pigmented nature of the lesion [21,22,23].

### Case Report Limitations

Due to the rarity of Nanta osteonevi and their inconspicuous gross appearance based on the literature, no gross clinical, dermatoscopic or gross pathological (during specimen sectioning) findings were obtained and hence cannot be included into the text to further expand its scope and relevance.

## 4. Conclusions

Osteonevus of Nanta is a rare phenomenon representing a combination of dermal melanocytic nevus with osteoid metaplasia. The condition is rare, predominantly developing in older females in the head and neck areas. While several mechanisms have been proposed for its development so far, the etiopathogenesis remains widely disputed.

## Data Availability

The original contributions presented in this study are included in the article. Further inquiries can be directed to the corresponding author.
